# Epidermal growth factor receptor (EGFR) is transcriptionally induced by the Y-box binding protein-1 (YB-1) and can be inhibited with Iressa in basal-like breast cancer, providing a potential target for therapy

**DOI:** 10.1186/bcr1767

**Published:** 2007-09-17

**Authors:** Anna L Stratford, Golareh Habibi, Arezoo Astanehe, Helen Jiang, Kaiji Hu, Eugene Park, Ashleen Shadeo, Timon PH Buys, Wan Lam, Trevor Pugh, Marco Marra, Torsten O Nielsen, Uwe Klinge, Peter R Mertens, Samuel Aparicio, Sandra E Dunn

**Affiliations:** 1 Laboratory for Oncogenomic Research, Department of Pediatrics, Child and Family Research Institute, University of British Columbia, Vancouver, British Columbia, Canada; 2 Department of Cancer Genetics and Developmental Biology, British Columbia Cancer Research Centre, Vancouver, British Columbia, Canada; 3 Michael Smith Genome Sciences Centre, British Columbia Cancer Agency, Vancouver, British Columbia, Canada; 4 Genetic Pathology Evaluation Centre of the Prostate Research Centre, Vancouver General Hospital and British Columbia Cancer Agency, Vancouver, British Columbia, Canada; 5 Department of Applied Medical Engineering, Helmholtz Institute, RWTH Aachen University, Aachen, Germany; 6 Departments of Nephrology and Clinical Immunology, University Hospital Aachen, RWTH Aachen, Germany; 7 Molecular Oncology and Breast Cancer Program, British Columbia Cancer Research Centre, Vancouver, British Columbia, Canada

## Abstract

**Introduction:**

Basal-like breast cancers (BLBCs) are very aggressive, and present serious clinical challenges as there are currently no targeted therapies available. We determined the regulatory role of Y-box binding protein-1 (YB-1) on epidermal growth factor receptor (EGFR) overexpression in BLBC, and the therapeutic potential of inhibiting EGFR. We pursued this in light of our recent work showing that YB-1 induces the expression of EGFR, a new BLBC marker.

**Methods:**

Primary tumour tissues were evaluated for YB1 protein expression by immunostaining tissue microarrays, while copy number changes were assessed by comparative genomic hybridization (CGH). The ability of YB-1 to regulate EGFR was evaluated using luciferase reporter, chromatin immunoprecipitation (ChIP) and gel shift assays. The impact of Iressa on monolayer cell growth was measured using an ArrayScan VTI high-throughput analyser to count cell number, and colony formation in soft agar was used to measure anchorage-independent growth.

**Results:**

YB-1 (27/37 or 73% of cases, *P *= 3.899 × 10^-4^) and EGFR (20/37 or 57.1% of cases, *P *= 9.206 × 10^-12^) are expressed in most cases of BLBC. However, they are not typically amplified in primary BLBC, suggesting overexpression owing to transcriptional activation. In support of this, we demonstrate that YB-1 promotes EGFR reporter activity. YB-1 specifically binds the EGFR promoter at two different YB-1-responsive elements (YREs) located at -940 and -968 using ChIP and gel shift assays in a manner that is dependent on the phosphorylation of S102 on YB-1. Inhibiting EGFR with Iressa suppressed the growth of SUM149 cells by ~40% in monolayer, independent of mutations in the receptor. More importantly anchorage-independent growth of BLBC cell lines was inhibited with combinations of Iressa and YB-1 suppression.

**Conclusion:**

We have identified for the first time a causal link for the expression of EGFR in BLBC through the induction by YB-1 where it binds specifically to two distinguished YREs. Finally, inhibition of EGFR in combination with suppression of YB-1 presents a potential opportunity for therapy in BLBC.

## Introduction

Identifying molecular targets for aggressive types of breast cancer is a milestone in the pursuit of individualized therapies. Gene-expression profiling of primary tumours has led to the following subcategories: luminal A, luminal B, the human epidermal growth factor receptor 2 (HER2) and the basal-like subtypes [1]. Our attention was drawn to the basal-like subtype, because these tumours do not respond to available targeted therapies and patients often die within two years of diagnosis [1,2]. Approximately 16% of all breast cancers are basal-like [3]; this corresponds to 46,400 women among the ~290,000 women in North America who will be diagnosed with breast cancer each year. What sets these tumours apart is that unlike many breast cancers, basal-like tumours do not express the estrogen receptor (ER) or progesterone receptor (PR), nor do they have amplified HER2. In the clinic, these tumours are often referred to as 'triple negative'. Women with triple negative tumours are not eligible for treatments that target ER (tamoxifen, aromatase inhibitors) or HER2 (trastuzumab). Instead they are treated with conventional chemotherapies, which have limited efficacy and many side effects. Therefore, it is critically important to identify alternative therapeutic strategies for basal-like breast cancer (BLBC).

We recently found that the transcription factor, Y-box binding protein-1 (YB-1), protein is commonly expressed in ER-negative breast cancers [4], and loss of this receptor is one of the hallmarks of BLBC [3,5]. More recently, YB-1 was pulled out of a screen from the BLBC cell line SUM149 in an attempt to identify genes that promote malignant transformation and tumour cell growth [6]. It has also been shown recently that epidermal growth factor receptor (EGFR) is highly expressed in approximately 50% of BLBCs [7]. Interestingly, YB-1 was originally isolated as a transcription factor that bound to enhancer sites on the *EGFR *gene, a finding that could explain, at least in part, why it promotes the growth of breast tumour cells [8]. In keeping with this possibility, Berquin *et al*. expressed YB-1 in mammary epithelial cells and observed a concomitant induction of EGFR [6]. We demonstrated in MCF-7 (ER-positive breast cancer cells) that overexpression of YB-1 leads to an increase in the levels of EGFR mRNA and protein [4]. This depends on the phosphorylation of YB-1 at S102 [4]. The YB-1 S102 site is located in the DNA-binding domain, suggesting that the effect on EGFR expression was likely to be through transcriptional regulation. We demonstrated that Akt binds directly to YB-1 and phosphorylates the S102 site, an observation that was subsequently confirmed in NIH3T3 cells [9]. We now believe that Akt is one of several kinases capable of phosphorylating the S102 site of YB-1. In support of this idea, inhibition of the kinase mTOR with rapamycin also inhibits YB-1 phosphorylation [9]. To understand this further, we demonstrated that YB-1 binds directly to the EGFR promoter within the first 1 kb of the transcription start site, and this occurs in a phosphorylation-dependent manner [4]. Consistent with these preclinical developments, we found that YB-1 is strongly correlated with EGFR in primary breast tumours by screening a tissue microarray of ~490 cases [4]. More recently, we have confirmed this observation in a cohort of ~2,222 primary breast tumours. In this study, YB-1 and EGFR are once again tightly correlated (*P *= 1.414 × 10^-24^; data not shown).

As both YB-1 and EGFR are expressed in BLBC, we questioned whether there was a relationship between these two genes in this particular subtype of breast cancer. First, we determined whether the overexpression was caused by gene amplification, and then further dissected the regulatory relationship between the two. Finally, we addressed whether inhibiting EGFR with Iressa (also referred to as ZD1839 or gefitinib) would slow the growth of BLBC.

## Materials and methods

### Tumour tissue microarrays and cluster analysis

Patients in this cohort and their tumours have been previously described [10], as have the staining conditions for YB-1, HER2, ER and PR [10]. EGFR and CK5/6 staining was performed according to Nielsen *et al*. [7]. In total, we had interpretable data on these proteins from 285/438 total breast cancer cases. For our analysis, YB-1 scored as 0 or 1 was considered negative, and 2 or 3 was considered positive. Data was filtered to exclude patients who were missing diagnostic or survival information. Results were considered statistically significant with *P *< 0.05. The data was analysed using SPSS software (Chicago, Illinois, USA).

### Comparative genomic hybridization

Ten formalin-fixed and paraffin-embedded archival BLBC cases from the Vancouver General Hospital archival TMA438 series were identified based on a distinct immunohistochemical (IHC) staining pattern (ER^-^, HER2^-^, PR^-^, CK5/6^+^). Tissue cores (1.5 mm) extracted from the source blocks were treated with xylene and ethanol, as described by Garnis *et al*. [11]. Samples were placed into DNA lysis buffer comprised of 10 mM Tris, 50 mM NaCl, 1 mM EDTA, 0.5% SDS placed at 55°C, and digested with proteinase K (Invitrogen, Carlsbad, California, USA) over a period of 48 to 72 h. DNA was extracted as previously described, RNase-treated, and quantified by ND-1000 Full Spectrum UV/Vis Spectrophotometer (Nanodrop, Wilmington, Delaware, USA) [11]. The ten BLBC specimens were assayed for genetic alterations using a whole-genome tiling path bacterial artificial chromosome (BAC) array in comparative genomic hybridization (CGH) experiments as previously described [12]. The submegabase resolution tiling set (SMRT) array contained 32,433 overlapping BACs-derived DNA segments that provide tiling coverage over the human physical genome map. All clones were spotted in triplicate, resulting in 97,299 elements over two sides. Hybridizations were scanned using a CCD-based imaging system (Arrayworx eAuto, Applied Precision; Issaquah, Washington, USA) and analyzed using SoftWoRx Tracker Spot Analysis software as previously described [13,14]. Data was filtered and breakpoints were identified as previously described by Baldwin *et al*. [15]. Clones with standard deviations between replicate spots of >0.075 and with signal-to-noise ratios of <3 were filtered from raw data. Genomic breakpoint boundaries were defined by aCGH-Smooth software and visual inspection. Log 2 signal intensity ratio thresholds were used to determine regions of gain and loss, with >0.5 representing a gain and <-0.5 representing a loss.

### Characterization of YB-1 and EGFR in basal-like breast cancer cells *in vitro*

184 htert cells were obtained from J. Carl Barrett at the US National Institute of Health, and were cultured as previously described [16]. SUM149 cells, selected because they express markers of BLBC [17,18], were purchased from Astrand (Ann Arbor, Michigan, USA) and were grown according to the manufacturer's recommendation. The cells were cultured in F-12 (Ham's) media (Gibco/Invitrogen, Burlington, Ontario, USA) supplemented with 5 μg/ml insulin (Sigma, Oakville, Ontario, Canada) 1 μg/ml hydrocortisone (Sigma), 10 mM HEPES (Sigma), 5% fetal bovine serum (Gibco/Invitrogen), and 100 units/ml of penicillin/streptomycin (Gibco/Invitrogen). MDA-MB-468 cells were obtained from the ATCC and cultured in Dulbecco's modified Eagle's medium, 10% FBS and 100 units/ml penicillin/streptomycin. HCC1937 breast cancer cells, also triple negative [19], were cultured in RPMI-1640 media supplemented with 5% FBS, 10 mM HEPES, 4.5 g/L glucose (Sigma), 1 mM sodium pyruvate (Sigma) and 100 units/ml penicillin/streptomycin. Cells were maintained at 37°C in 5% CO_2 _and passaged every 2 days.

Proteins were isolated from log growing 184 htert, SUM149 and HCC1937 cells using an ELB buffer [4]. YB-1, EGFR and actin were detected by immunoblotting. The YB-1 polyclonal antibody (courtesy of Colleen Nelson, University of British Columbia, Vancouver, Canada) was used at a dilution of 1:10,000. The EGFR monoclonal (clone 6F1, StressGen, San Diego, California, USA) and actin (Sigma, St Louis Missouri, USA) antibodies were diluted 1:1000.

### Chromatin immunoprecipitation

SUM149 cells were plated at a density of 1 × 10^7 ^in a 150 mm dish and YB-1-promoter complexes were isolated by chromatin immunoprecipitation (ChIP) as previously described [4]. The primers to each of the potential YB-1 binding sites were previously described [4]. The EGFR promoter was amplified (40 cycles) using primers that span regions within the first 2 kb upstream of the start site. The input DNA was diluted fourfold before amplification.

### Serial ChIP to determine YB-1 phosphorylation status

To determine whether YB-1 is serine phosphorylated at the EGFR promoter, complexes were isolated as described above with the chicken YB-1 antibody and then eluted by incubation in 10 mmol/L DTT at 37°C for 30 min with agitation. The eluate was diluted 1:50 with buffer (20 mmol/L Tris (pH 8.1), 150 mmol/L NaCl, 2 mmol/L EDTA, and 1% Triton X-100) and re-immunoprecipitated with 5 μg of anti-phosphoserine antibody (StressGen) overnight at 4°C. Secondary immunocomplexes were incubated with salmon sperm DNA/protein A agarose for 2 h at 4°C. Subsequent steps followed the ChIP protocol described previously by [4] and PCR was performed with primers to the EGFR 2a site as described above. To test for non-specific binding species, matched IgY and IgG were incubated with an equal amount of SUM149 cross-linked DNA. The sample was then processed as described above and amplified with primers to EGFR 2a. The input DNA was also introduced as a positive control.

ChIP was also performed using a phospho-YB-1 (S102) antibody (in collaboration with Peter Mertens, Germany). The peptide sequence and supportive data demonstrating the specificity of the antibody was recently described by us [20]. The immunoprecipitation was carried out as described above for YB-1 with protein G-agarose used in place of PreciPhen beads and rabbit IgG instead of IgY.

### Electrophoretic mobility shift assay (EMSA)

Nuclear and cytoplasmic protein was extracted from log-growing SUM149 cells, MDA-MB-468 or HCC1937 cells using the NE-PER nuclear and cytoplasmic extraction reagents (Pierce Biotechnology, Rockford, Illinois, USA) following the manufacturer's protocol. Briefly, cells were centrifuged to obtain a packed cell volume and lysed in ice cold CER I with protease inhibitors. Following 5 min on ice, ice-cold CER II was added and samples centrifuged at 13,000 *g *for 10 min. Cytoplasmic protein was retained and the pellet re-suspended in ice-cold NER with protease inhibitors. The sample was incubated on ice for 40 min with frequent mixes and then centrifuged at 13,000 *g *for 10 min. The supernatant containing nuclear protein was stored. Proteins were quantified using the Bradford Assay. EMSAs were carried out using the Lightshift Chemiluminescent EMSA kit (Pierce Biotechnology), following the manufacturer's protocol. 5' Biotin-labelled complementary oligonucleotides with the following sequences, wild-type (-979 to -937) TTCACACATTGGCTTCAAAGTACCCATGGCTGGTTGCAATAAACAT, -968 mutant 5'-TTCACAC*CCCC*GCTTCAAAGTACCCATGGCTGGTTGCAATAAACAT, -940 mutant 5'-TTCACACATTGGCTTCAAAGTACCCATGGCTGGTTG*CCCC*AAACAT and double mutant 5' -TTCACAC*CCCC*GCTTCAAAGTACCCATGGCTGGTTG*CCCC*AAACAT were annealed to form double stranded DNA. Binding reactions consisted of 1 × binding buffer, 50 ng/μl poly dIdC, 20 fmol Biotin-labeled DNA and 5 μg nuclear protein in a 20 μl reaction. Competition reactions included 16 pmol unlabelled oligonucleotide (800-fold excess), and 1 μg chicken anti-YB-1 antibody was included to determine YB-1 involvement. An antibody to CREB (1 μg) was introduced as a negative control. The protein was incubated with the unlabelled oligonucleotide or the antibody for 20 min before the addition of the biotin-labelled oligonucleotide. The samples were incubated for 20 min at room temperature. The reaction mixture was run on a 6% non-denaturing polyacrylamide gel and transferred to a positively charged nylon membrane (Amersham Biosciences, Little Chalfont, UK). DNA was crosslinked to the membrane at 120 mJ/cm^2 ^using a UV-light crosslinker (Stratalinker, Stratagene, La Jolla, California, USA) and detected using chemiluminescence (Pierce Biotechnology).

### Nuclear extraction of primary BLBC tumours

Tissue slices from six BLBC tumour specimens were obtained from the British Columbia Cancer Agency, Canada. Nuclear fractions were extracted using the NE-PER nuclear and cytoplasmic extraction reagents as described above. Since tissue was limited the samples were pooled before the nuclear extraction step. Electrophoretic mobility shift assays were carried out as described above with 10 μg protein.

### EGFR luciferase assay

To determine whether YB-1 has a direct effect on EGFR promoter activity the normal breast cell line, 184 htert, was transfected with a 1 kb EGFR promoter construct [21] (courtesy of Alfred C Johnson US National Cancer Institute, Massachusetts, USA), a renilla expression vector, pRL-TK (Promega, Madison, Wisconsin, USA), and a YB-1 expression plasmid, a YB-1 S102 mutant (A102) or empty vector. The cells were plated in 6-well plates (4 × 10^5 ^cells/well) and transfected with a total of 1.5 μg DNA using lipofectamine 2000 (Invitrogen). Cells were harvested 24 h post-transfection in 1 × PLB buffer (Promega), and luciferase activity measured. All luciferase measurements were normalized to the renilla reading from the same sample. To carry out the inverse experiment the Fast-Forward Protocol provided with the HiPerFect Transfection Reagent (Qiagen, Mississauga, Ontario, USA) was used to achieve knockdown of YB-1 in SUM149 and HCC1937 cells using small interfering RNA (siRNA) (for control and YB-1 siRNA sequences see [4]). Briefly, cells were seeded at 4 × 10^5 ^cells/well of a 6-well plate in 2 ml media shortly before transfection. siRNA was diluted to 100 μl in serum-free media to achieve a final concentration of 5 nM (SUM149) or 20 nM (HCC1937), and 3 μl HiPerFect was added. Samples were vortexed, incubated at room temperature for 10 min, and then added drop-wise to the cells. At 48 h the cells were re-plated in 6-well plates (4 × 10^5 ^cells/well and transfected with the pER1, pRL-TK and empty vector and harvested at 24 h post-transfection as described above. Cell lysates were also collected at the time of re-plating to ensure successful knockdown of YB-1. The experiments were performed in triplicate on two separate occasions. The results are reported as the average of two experiments.

### Cell viability following treatment with Iressa

SUM149 breast cancer cells were plated in 96-well plates (5 × 10^3 ^cells/well) and incubated for 24 h at 37°C in the growth media described above. Cells were treated with Iressa (isolated from tablets purchased from Astra Zeneca and kindly provided by Ching-Shih Chen, Ohio State University, USA) at the following concentrations; 0, 0.25, 0.5, 1 and 2 μM with dimethyl sulphoxide (DMSO) as vehicle control. Cell number was ascertained after 72 h treatment. Cells were washed in PBS and then incubated with Hoechst dye (1 μg/ml) for 15 mins. Nuclei counts/well were determined using the ArrayScan VTI high throughput analyser. Statistical analyses were carried out using the Student *t *test with significance accepted when *P *< 0.05.

### Growth in soft agar

SUM149 cells were plated at a density of 2.5 × 10^4 ^in a 24-well plate in 0.6% agar, as previously described [10] and supplemented with Iressa in the cell layer (concentrations as above). HCC1937 cells were treated with CTRL and YB-1 siRNA for 48 hours and then plated at a density of 10 × 10^3 ^in 0.6% agar. At the time of seeding the agar was supplemented with Iressa (0.25 to 2 μM) as described earlier. Colonies developed over 30 days and were then counted. Each experiment was performed in replicates of four and repeated twice.

### EGFR sequencing from SUM149 cells

Genomic DNA was isolated from 2 × 10^7 ^SUM149 cells using phenol chloroform extraction followed by alcohol precipitation (modified from [22]). DNA was quantified in a UV spectrophotometer. EGFR exons 1 to 28 were amplified by PCR and sequenced using standard techniques used by the British Columbia Cancer Agency Michael Smith Genome Sciences Centre. PCR primers were designed using human genome reference sequence acquired from the UCSC Genome Browser [23] ([24]) and the Primer3 program [25]. The PCR primer sequences are listed in Additional file 1. Each PCR reaction was performed on 10 ng of SUM149 DNA and the products were visualized on a 2% agarose gel. PCR products were cleaned up using Ampure magnetic beads (Agencourt, Beverly, Massachusetts, USA) and sequenced using a standard BigDye Terminator v3.1 cycle sequencing chemistry and Applied Biosystems (Foster City, California, USA) 3730 × l DNA Analyzer.

## Results

### YB-1 and EGFR amplification is not common in BLBC, indicating changes in transcriptional control

**Figure 1:**
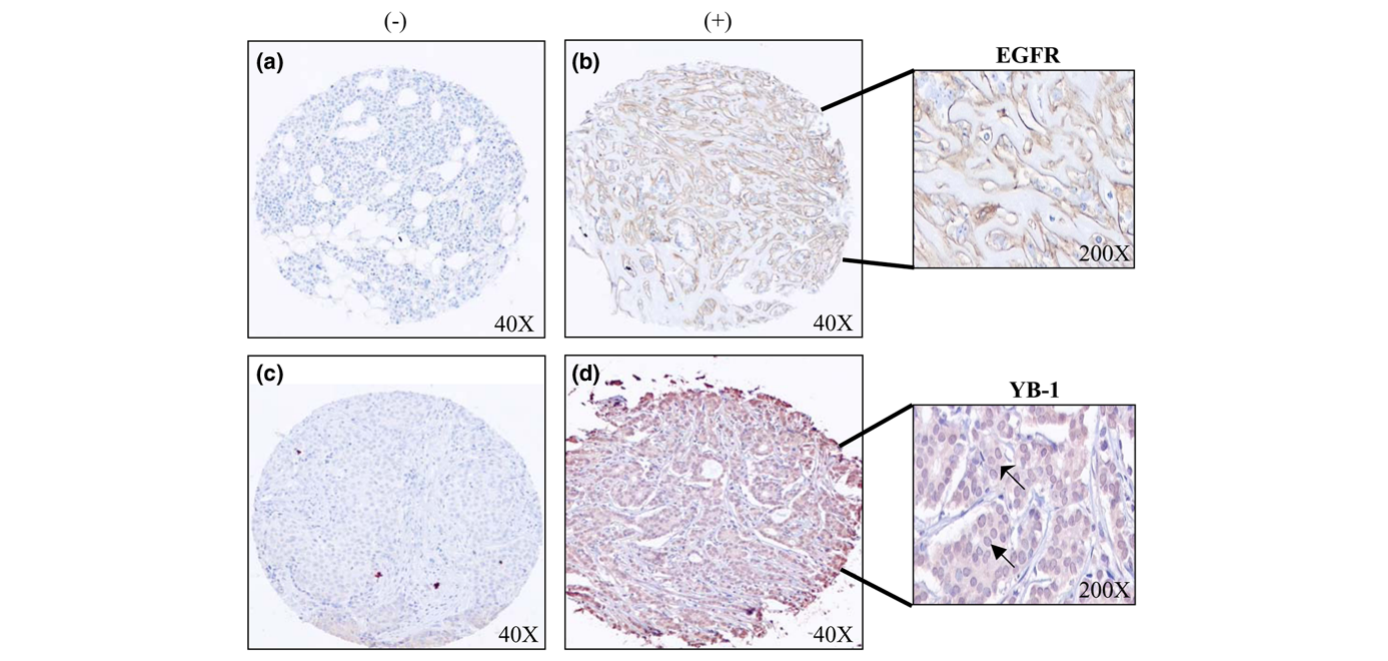
Epidermal growth factor receptor (EGFR) and Y-box binding protein 1 (YB-1) are detected in basal-like breast cancer specimens on a tumour tissue microarray. (a) EGFR-negative staining (40×). (b) Brown cells indicate EGFR positivity (40×), a segment of the core is magnified at 200×. (c) YB-1-negative staining (40×). (d) Brown staining indicates YB-1 positivity (40×), which is detected in both the nucleus and cytoplasm (arrowheads 200×).

**Figure 2:**
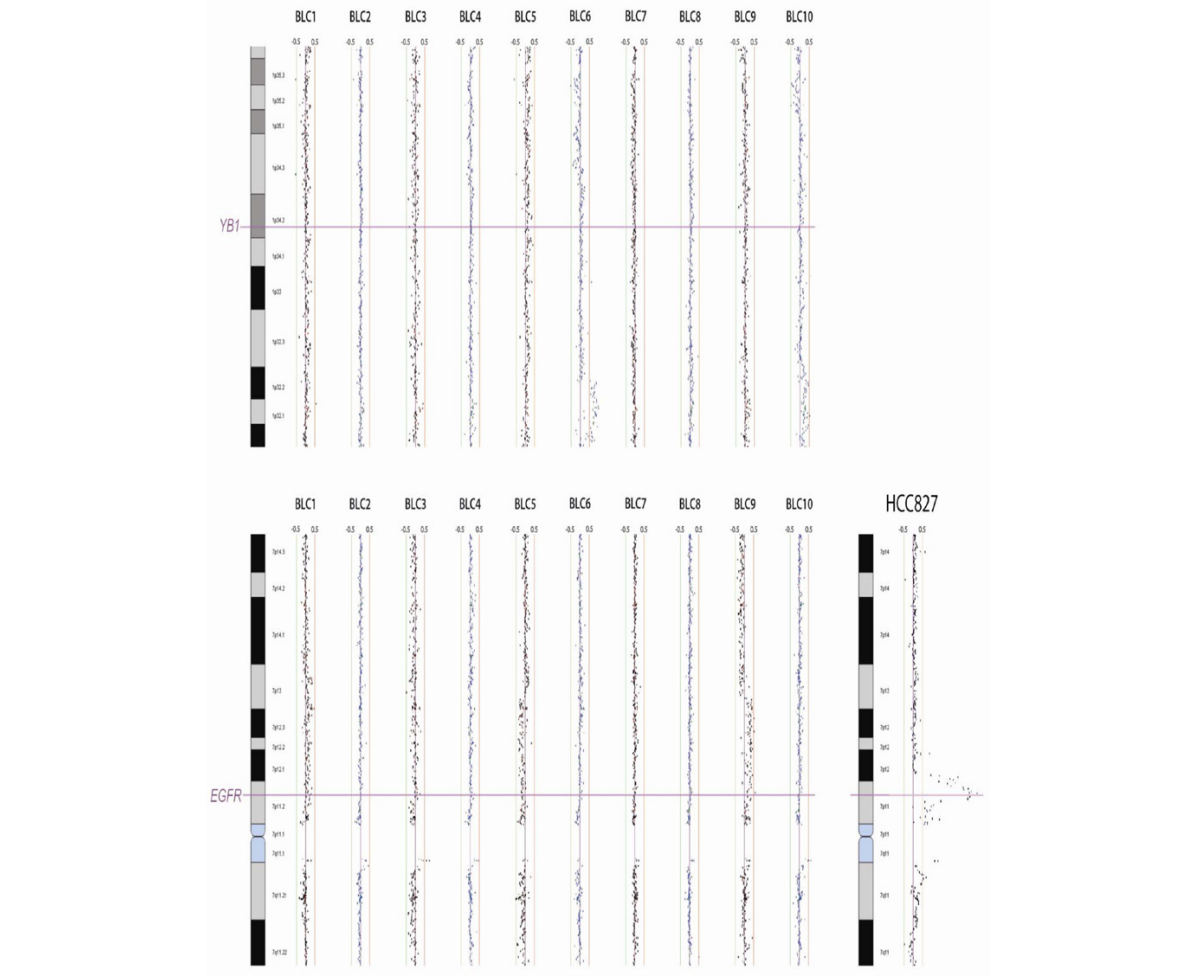
Basal-like breast tumours do not exhibit amplifications for epidermal growth factor receptor (EGFR) or Y-box binding protein 1 (YB-1). Primary breast tumours were evaluated for genetic amplifications using SMRT array CGH. DNA was isolated from ten primary basal-like breast tumours and genomic profiles were generated by submegabase resolution tiling array comparative genomic hybridisation. There was no obvious gain of copy number on chromosomes 1 or 7, representing the loci for YB-1 and EGFR, respectively. The exception to this trend was BLC9, where there was a large amplicon on chromosome 7. The lung adenocarcinoma cell line HCC827 was included as a positive control of EGFR amplification.

Breast tumour tissue microarrays were profiled to evaluate the frequency to which EGFR and YB-1 are expressed in triple negative breast cancers. Such tumours express YB-1 and EGFR in 73% and 57.1% of the BLBC cases, respectively (Table 1). Representative immunohistochemical images for both EGFR and YB-1 are shown in Figure 1. As indicated by the arrowheads, YB-1 was expressed in the cytoplasm as well as the nucleus. Although we have established that YB-1 and EGFR are frequently expressed in triple-negative breast cancers, it is not clear why this occurs. One possibility is that these genes are both amplified during the development of BLBC. To study this, we isolated DNA from 10 primary BLBCs and evaluated them for copy number changes by array CGH using a genome-spanning tiling path array (SMRT) [26]. Copy number changes were not observed at the *YB-1 *locus (1p34.2) or the *EGFR *locus (7p13-11.2) in 10/10 and 9/10 cases, respectively (Figure 2). A borderline 10 Mb segmental gain was present in one (referred to as BLC9) of the 10 cases at 7p13-11.2 encompassing many gene loci including *EGFR *(Figure 2). The lung cancer adenocarcinoma cell line (HCC827), known to have amplified EGFR, was evaluated as a positive control (Figure 2). Overall neither *YB-1 *nor *EGFR *were commonly amplified, suggesting expression is increased owing to promoter activation.

**Table 1:** YB-1 is highly expressed in triple negative breast cancer

Marker	Correlation	Likelihood ratio value
YB-1	*P *= 3.899 × 10^-4^	12.58
	*N *= 27/37 (73%)	
EGFR	*P *= 9.206 × 10^-12^	46.491
	*N *= 20/37 (57.1%)	

Y-box binding protein 1 (YB-1) is expressed in 73% of triple negative breast cancers in the TMA438. Epidermal growth factor receptor (EGFR) is expressed in 57.1% of these cases.

### YB-1 regulates the expression of EGFR in BLBC

**Figure 3:**
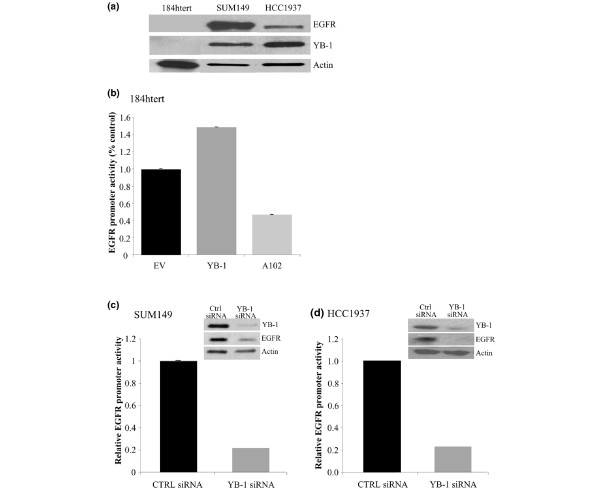
Y-box binding protein 1 (YB-1) regulates the expression of epidermal growth factor receptor (EGFR) in basal-like breast cancer cells. (a) The levels of YB-1 and EGFR proteins were compared between immortalized breast epithelial cells, 184 htert, SUM149 and HCC1937 basal-like breast cancer cells. Actin was evaluated as a control for equal protein input. (b) 184 htert cells were transfected with an EGFR promoter (1 kb) luciferase construct (pER1), a control renilla plasmid (pRL-TK) and either flag-EV or flag-YB-1 or flag-YB-1(A102). Luciferase and renilla activity were measured after 24 hours. YB-1 induced EGFR promoter activity by 1.5-fold (*P *= 0.04, *N *= 6), whereas the A102 mutant did not. (c) SUM149 cells were treated with YB-1 small interfering RNA (siRNA) (5 nM) for 48 h. The cells were then transfected with the EGFR reporter for 24 h and compared with the empty vector. Loss of YB-1 expression resulted in a 78% decrease in EGFR reporter activity (*P *= 4.53 × 10^-5^, *N *= 6). Inset: evidence that siRNA targeting YB-1 causes a decrease in expression of the protein. Actin was used as a loading control. (d) The same experiment was repeated using HCC1937 cells treated with 20 nM YB-1 siRNA for 48 h. Loss of YB-1 expression resulted in a 77% reduction in EGFR promoter activity (*P *= 5.98 × 10^-7^, *N *= 6).

To perform functional investigations into the role of YB-1 and EGFR in BLBC, we tested the SUM149 and HCC1937 cell lines, which have a basal phenotype [17-19,27]. Initially the levels of YB-1 and EGFR were compared between 184 htert (immortalized breast epithelial cells) and the cancer cells. SUM149 and HCC1937 cells had high levels of YB-1 and EGFR compared with the 184 htert cells (Figure 3a). Building on the observation that YB-1 binds to the EGFR promoter within the first 1 kb of the start site [4], we then investigated whether there was a causal link between YB-1 and the expression of EGFR in the SUM149 and HCC1937 cells. First, we have determined that YB-1 was able to stimulate EGFR promoter activity using a luciferase reporter construct containing the first 1 kb of the EGFR promoter. Immortalized breast cells (184 hterts) confirmed not to express YB-1 (Figure 3a) transfected with a hYB-1 plasmid increased EGFR luciferase activity 1.5-fold compared with the control cells (*P *= 0.04, *N *= 6) (Figure 3b). Interestingly, when cells were transfected with the YB-1 mutant (A102) that could no longer be phosphorylated at S102, there was a significant attenuation in reporter activity compared with control cells (*P *= 0.013, *N *= 6) (Figure 3b). We then addressed whether silencing the high levels of YB-1 in the SUM149 and HCC1937 cells would attenuate EGFR reporter activity. YB-1 was knocked down with siRNA for 48 h and then transfected with the EGFR reporter. Under these conditions, we observed a 78% and 77% loss in EGFR reporter activity in SUM149 and HCC1937 cells, respectively (*P *= 4.53 × 10^-5 ^and *P *= 5.98 × 10^-7^, *N *= 6) (Figure 3c,d). Therefore, through gain-of-function and loss-of-function studies we showed that YB-1 transactivates the EGFR promoter, and that this occurs in a manner that is dependent on the S102 DNA binding site.

**Figure 4:**
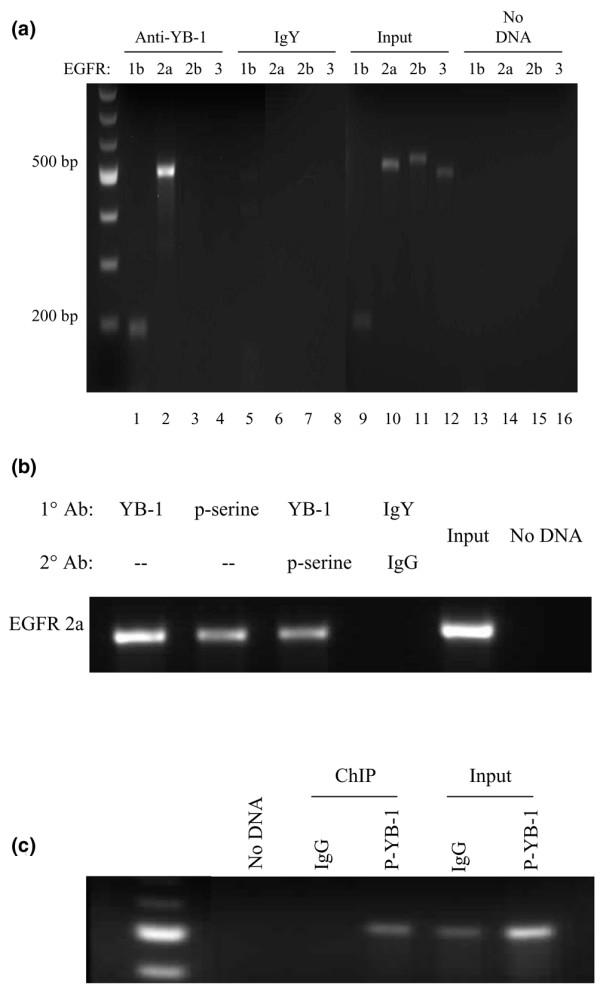
Y-box binding protein 1 (YB-1) binds to the epidermal growth factor receptor (EGFR) promoter. (a) Chromatin immunoprecipitation was performed on SUM149 cells. YB-1 binds to the EGFR promoter in the basal-like cells where the 2a loci is the preferred binding site (lane 2). Weak binding was also detected with the 1b primers (lane 1). No binding was observed in the 2b or 3 sites (lanes 3 to 4), nor was there any non-specific binding detected in the IgY negative controls (lanes 5 to 8). Input DNA was diluted fourfold and amplified to demonstrate that the primer produced an expected product (lanes 9 to 12). The no input controls (lanes 13 to 16) are presented to show a lack of non-specific amplifications. (b) Serial ChIP was performed by sequentially pulling down YB-1 and then immunoprecipitating with a phospho-serine antibody. This demonstrated that at least some of the YB-1 is serine phosphorylated when bound to the EGFR 2a site. YB-1 binds to the 2a site (lane 1) as expected. Similarly, the phospho-serine antibody pulls down a complex that can be amplified with the 2a primers (lane 2). Re-ChIP with the YB-1 antibody and subsequently with the phospho-serine antibody also bound to EGFR at the 2a site (lane 3). A phospho-serine YB-1 complex bound to the 2a site on EGFR (lane 3). Species-matched IgG and IgY controls were included to show that the binding was specific (lane 4). The input DNA and no DNA controls were also included (lanes 5 and 6). (c) ChIP was carried out using a phospho-YB-1 antibody (S102), and binding was detected for the EGFR 2a region (lane 4). There was no binding observed when immunoprecipitation was performed using IgG as a control (lane 3). Input DNA was diluted fourfold and amplified to demonstrate that the primer produced an expected product (lanes 5 and 6). Lane 1 is the DNA ladder.

Having demonstrated that YB-1 can transactivate EGFR we next determined whether YB-1 interacted with the EGFR promoter in the basal-like breast cancer cells to further confirm binding observed in breast cancer cell lines that were not basal-like [4], and to address whether this occurs in a manner that is dependent on S102 phoshorylation using a newly developed antibody directed at YB-1(S102) [20]. Using the primer sets previously described [4] we show that, in SUM149 cells, YB-1 binds to the EGFR promoter within the first 1 kb, and most strongly at the 2a site (Figure 4a, lane 2). This interaction is also observed in the basal-like MDA-MB-468 cells that we have previously reported [20]. Binding did not occur in the SUM149 cells in the regions designated 2b and 3 (Figure 4a, lanes 3 and 4). We confirmed that binding was specific and did not bind to the IgY alone (Figure 4a, lanes 5 to 8), and that the primers could amplify genomic input DNA (Figure 4a, lanes 9 to 13) compared with the negative controls in which no DNA was added to the amplification reaction (Figure 4a, lanes 13 to 16). This binding pattern is in keeping with our previous work showing that YB-1 binds to the EGFR promoter within the first 1 kb in a manner that was dependent on phosphorylation at S102 [4]. As the phosphorylation status of YB-1 affected its ability to transactivate EGFR, we assessed whether this was also the case in the interaction between the YB-1 and 2a site of the promoter. We therefore questioned whether YB-1 is serine phosphorylated when it binds to the 2a site. To address this, we initially developed serial ChIP protocol, whereby YB-1 was initially used to pulldown protein–DNA complexes, and the resulting samples were then immunoprecipitated with an antibody to phospho-serine. Using this method we were able to show that YB-1 is serine phosphorylated when it binds to the 2a site (Figure 4b). More recently, we have had the opportunity to test a new polyclonal antibody raised against YB-1(S102) specifically [20]. In this case, binding to the 2a site is also observed (Figure 4c) further supporting the idea that YB-1 is serine phosphorylated at S102 when it binds to the EGFR promoter.

**Figure 5:**
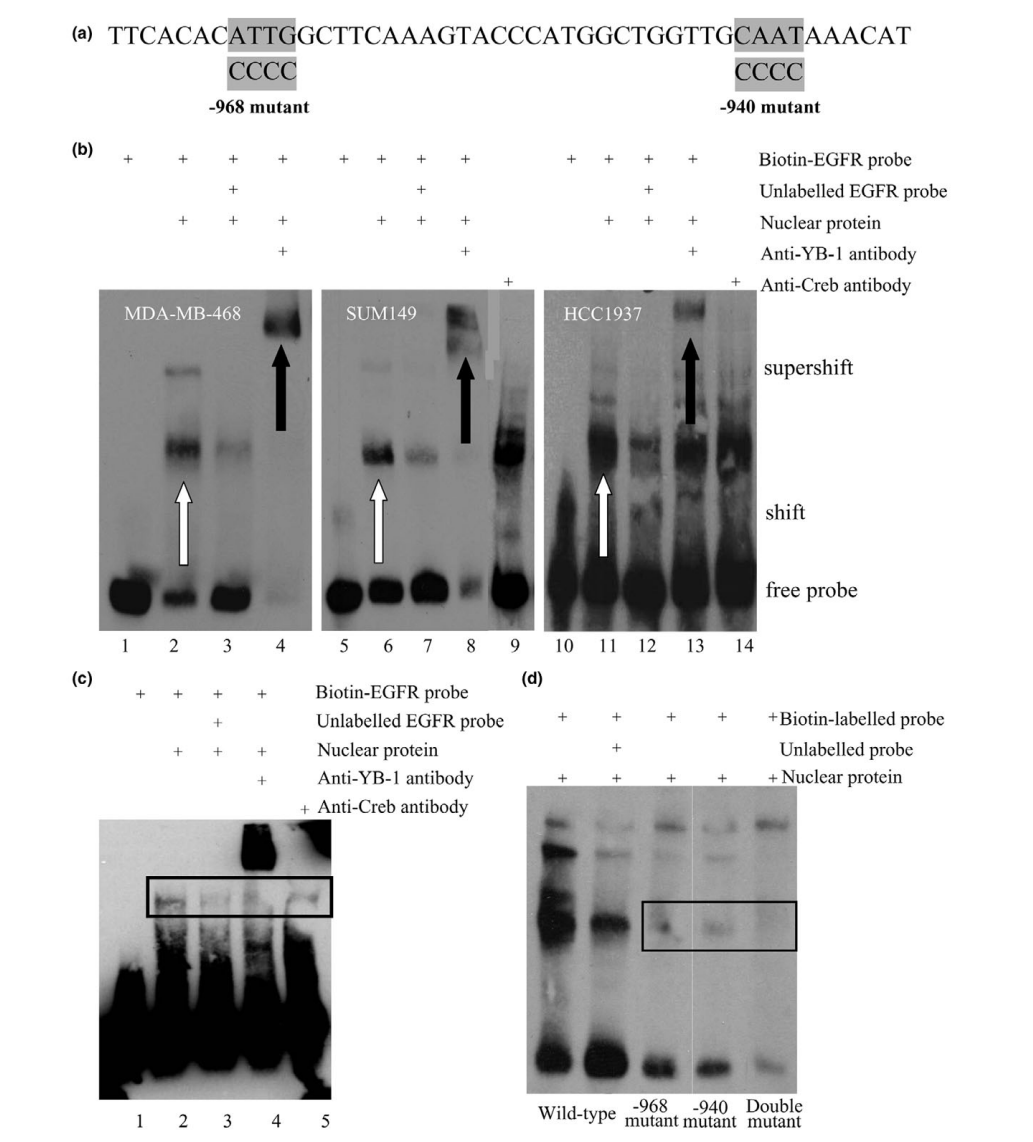
Y-box binding protein 1 (YB-1) binds to specific sites within the epidermal growth factor receptor (EGFR) promoter. (a) Sequence of the EGFR2a oligonucleotide used in the gel shift assays (-979 to -934). Highlighted sequences are the potential YB-1 binding sites. The substitutions made in the two mutants are given under the wild-type sequence. (b) Direct evidence for YB-1 binding to the EGFR promoter using gel shift assays. Nuclear extract from SUM149, MDA-MB-468 or HCC1937 cells were incubated in the presence of the EGFR oligonucleotide spanning -979 to -934. There was no binding in the absence of protein (lanes 1, 5 and 10), whereas the addition of the nuclear extract (lanes 2, 6 and 11) resulted in strong binding that could be inhibited with the unlabelled oligonucleotide (lanes 3, 7 and 12). The addition of a YB-1 antibody caused a supershift (lane 4, 8 and 13) that did not occur when the non-related CREB antibody was used (lanes 9 and 14). (c) Nuclear extracts from 6 primary BLBC samples were pooled and used in a gel shift assay for the EGFR 2a site. Lane 1 contains EGFR2a biotin-labelled oligo only. Binding to the probe is evident in lane 2, which was competed off in lane 3 and supershifted with a YB-1 antibody in lane 4. A CREB antibody was used to demonstrate specificity of the supershift (lane 5). (d) Validation of putative YB-1-responsive elements on the EGFR promoter. SUM149 nuclear extracts were incubated with either wild-type (lane 1) or mutant biotin oligo nucleotides (lanes 3, 4, and 5). A competition reaction was carried out against the wild-type (lane 2). nuclear extract bound to the wild-type sequence (lane 1), but was unable to bind the mutants (lanes 3, 4 and 5).

The ability of YB-1 to bind to the EGFR promoter specifically at the 2a region was further confirmed using gel shift assays. Nuclear extracts from SUM149, MDA-MB-468 and HCC1937 cells were incubated with a biotin-labelled oligonucleotide probe spanning -979 to -934 of the EGFR promoter (Figure 5a). MDA-MB-468 and HCC1937 cells were used as an additional basal-like cancer cell lines as they are triple negative and they overexpress EGFR. Compared with the unbound probe (Figure 5b, lanes 1, 5 and 10), the introduction of the nuclear extract from all cell lines produced intense binding to the EGFR promoter (Figure 5b, lanes 2, 6 and 11) that could be competitively inhibited with unlabelled probe (Figure 5b, lanes 3, 7 and 12). Co-incubation of the nuclear extract with a YB-1 antibody caused a supershift (Figure 5b, lanes 4, 8 and 13), an effect not observed when an unrelated CREB antibody was used in the same reaction (Figure 5b, lanes 9 and 14); therefore, we validated our ChIP results by demonstrating that YB-1 binds directly to the EGFR promoter. We have also been able to show that YB-1 binds to the 2a region of the EGFR promoter in primary BLBC cancer samples (Figure 5c, lane 2). This interaction could be competed off with unlabelled oligo (Figure 5c, lane 3) and supershifted using the YB-1 antibody (Figure 5c lane 4). To further dissect YB-1 binding within the 2a region we designed biotin-labelled oligonucleotides in which the YB-1-responsive elements (YREs) were mutated at -968, -940 or both sites (Figure 5a). Losing either of the YREs resulted in less YB-1 binding compared with the wild-type EGFR promoter sequence (Figure 5d). These data verify that the -968 and -940 binding sites are bona fide YREs. Together these data show that YB-1 is able to bind to the first 1 kb of the EGFR promoter, and this leads to transactivation in a phosphorylation dependent manner.

### Inhibiting EGFR suppresses the growth of BLBC cells

**Figure 6:**
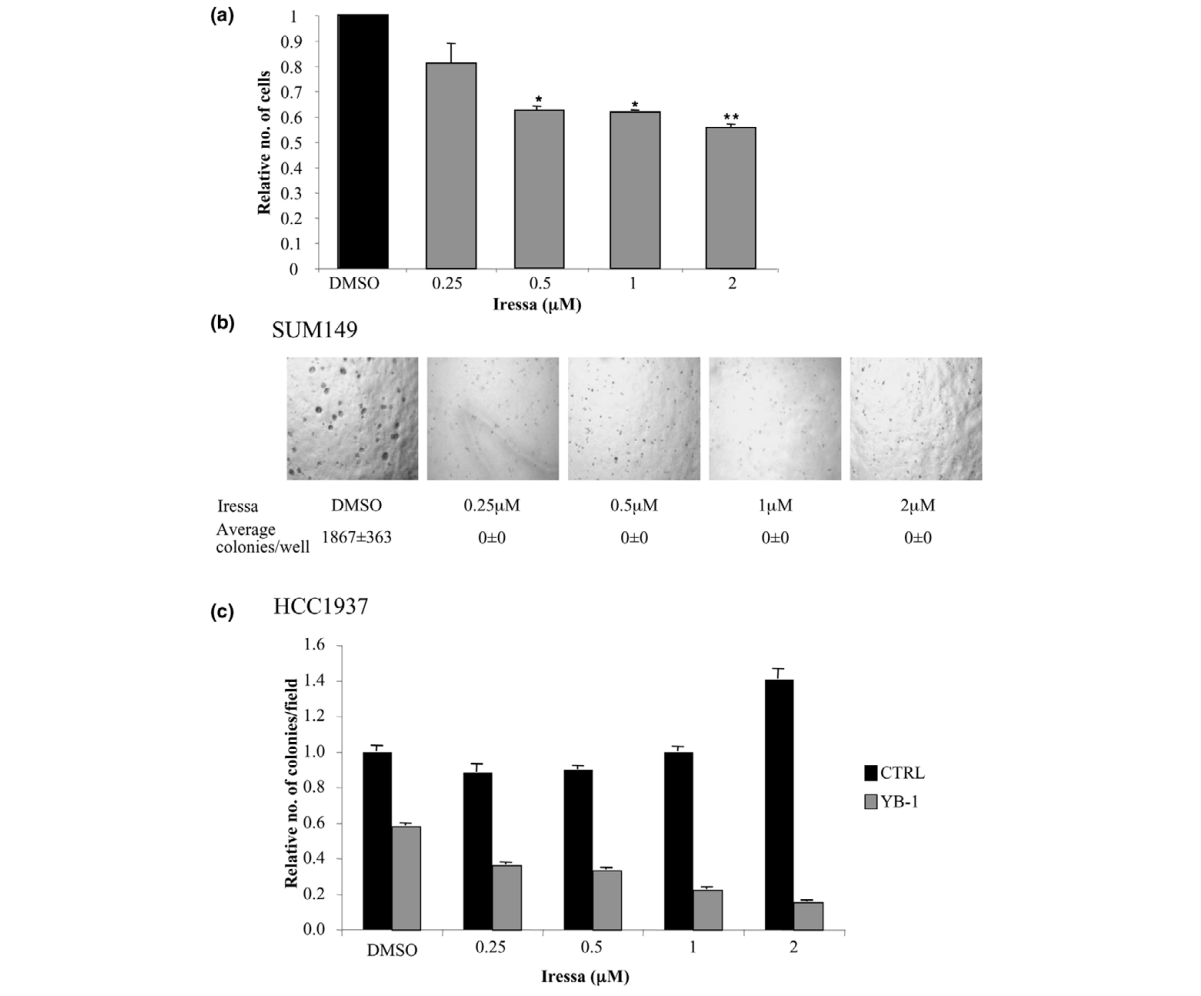
Inhibiting epidermal growth factor receptor (EGFR) suppresses the growth of basal-like breast cancer cells. (a) Inhibition of EGFR with Iressa (0.25, 0.5, 1 and 2 μM) blocks the growth of basal-like breast cancer cells by up to 40% when the cells were treated for 72 h (0.5 μM *P *= 0.02, 1 μM *P *= 0.02, 2 μM *P *= 0.07). Each experiment was performed in replicates of six on two separate occasions. (b) Anchorage-independent growth was measured by counting colonies formed after 4 weeks exposure to Iressa or vehicle control. Representative images of colonies following each treatment are shown, with average colony number/well shown underneath. The ability to form colonies was completely lost in the presence of concentrations of Iressa as low as 0.25 μM in SUM149 cells. (c) The ability of HCC1937 cells to form colonies was not effected by Iressa alone; however, knockdown of YB-1 significantly reduced the number of colonies (*P *< 0.001). The addition of Iressa further reduced the number of colonies. This was highly significant at all concentrations (*P *< 0.001). Statistical analysis carried out using students *t*-test; *P < 0.05, **P < 0.01.

As there are several commercially available EGFR inhibitors available (such as Iressa and erlotinib), we questioned whether targeting this receptor tyrosine kinase would be effective in cells in which it is highly expressed. Monolayer cell growth could be inhibited by up to 40% when SUM149 cells were treated with Iressa (0 to 2 μM) for 72 h (Figure 6a); however, more interestingly, if we grew SUM149 cells in anchorage-independent conditions then formation of colonies, and therefore the ability of the cells to transform, was completely abolished in the presence of as little as 0.25 μM Iressa compared with vehicle-treated cells (control 1,867 ± 363, 0.25 to 2 μM Iressa 0 ± 0) (Figure 6b). These concentrations are achievable in patients [28] and have previously been shown to inhibit MAP kinase signalling [29]. To confirm this observation, we also found that low doses of Iressa inhibited signalling through the MAP kinase pathway (data not shown). To ascertain whether this sensitivity was inherent to other BLBC cell lines we repeated the same experiment in HCC1937 cells, and somewhat surprisingly these cells were still able to form colonies in anchorage-independent conditions in the presence of up to 2 μM Iressa. Similarly, the MDA-MB-468 basal-like breast cancer cells are insensitive to Iressa initially but can be sensitized by targeting PI3 kinase with LY294002 [30]; an observation that we independently confirmed (data not shown). In a separate study, LY294002 has been shown to inhibit phosphorylation of YB-1 [9]. This is in keeping with our previous studies demonstrating that YB-1 is phosphorylated by Akt in response to PI3 kinase activation [10]. We therefore questioned whether knocking down YB-1 in HCC1937 cells before treating with Iressa would be effective at reducing the ability of these cells to grow in soft agar. The suppression of YB-1 alone caused a 42% reduction in the number of colonies compared with control (*P *= 0.0008), but there was further significant decreases in colony number with the addition of as little as 0.25 μM Iressa (*P *< 0.001 for all concentratons)(Figure 6c). Thus, our studies indicate that although some BLBC cells may be sensitive to Iressa, for others the inhibition of YB-1 may be necessary to sensitize the cells to drug.

We were rather surprised that the SUM149 cells were so sensitive to the drug. An obvious explanation would be that these cells express activating mutations in EGFR that would make them sensitive to Iressa, as has been described for lung cancer [31]. We therefore sequenced EGFR but unexpectedly did not find such mutations. All 28 exons coding for this gene were amplified by PCR and sequenced. Activating mutations such as L858R or delL747-P753insS that have previously been reported to be associated with Iressa sensitivity [31] were not found. However, we did identify five single-nucleotide polymorphisms (SNPs) in exons 12, 13, 15 and 20 (Additional file 2). There was one homozygous non-translated SNP (rs712830), three heterozygous synonymous SNPs (rs17290005, rs17290162 and rs17337198), and one heterozygous non-synonymous SNP (rs11543848). These dbSNPs have been previously identified for EGFR ([32]), although their functional significance is not yet known. The SNP of most interest is R521K, located on exon 13, because it results in an amino acid change located in the extracellular domain of the receptor [33].

We concluded that irrespective of 'activating mutations' in EGFR, Iressa inhibits the growth of basal-like breast cancer cells. In some cases, co-targeting EGFR and YB-1 may be necessary to optimally inhibit the growth of these aggressive breast cancer cells. Given these data, we concluded that inhibiting EGFR and YB-1 significantly slows the growth of BLBC cells.

## Discussion

It has previously been reported that both YB-1 and EGFR are highly expressed in aggressive forms of breast cancer [4,7]. In this study we show that although these proteins are a feature of BLBC, neither gene is overexpressed owing to amplification. In further studying YB-1 as a transcription factor, we show that it transcriptionally induces EGFR in basal-like cell lines, which could lead to the increased expression observed. Importantly, we have been able to pinpoint that YB-1 binds specifically to YREs located at -968 and -940. On precisely identifying the bona fide YREs on the EGFR promoter, we demonstrate for the first time that binding to this region occurs when YB-1 is phosphorylated at S102. The high levels of both EGFR and YB-1 in BLBC begs the question of whether either of them are potential therapeutic targets. Based on the poor survival rates previously reported [1,2] it is clear that the BLBC subtype represents a very aggressive form of the disease, and EGFR is a rational target for the treatment of BLBC. In fact, since it was reportedly associated with this subtype of breast cancer in 2004 [7], the use of EGFR in classifying basal-like tumours by immunohistochemistry has become widely accepted [34,35].

We show for the first time that the EGFR inhibitor Iressa suppresses the growth of SUM149 cells, a model for BLBC, *in vitro *at concentrations achievable in patients [28]. This is not the case for other BLBC models, as no inhibition of anchorage-independent growth was evident in the HCC1937 cells when they were treated with Iressa alone. This insensitivity is also reported in MDA-MB-468s [30] and MDA-MB-231 cells, another triple negative cell line with high levels of EGFR expression [36,37]. Why the SUM149 cells alone are sensitive to the drug is not clear. Several studies suggest that activating mutations in EGFR are predictive of whether inhibitors, such as Iressa, would be effective in patients with lung cancer [31,38]. The same could be true for breast cancer, but it is not known whether BLBCs harbour such mutations. However, we did sequence the entire *EGFR *gene from SUM149 cells and did not find activating mutations previously described for lung cancer. Whether the SNP at R521K influences sensitivity to Iressa is not known, and warrants further investigation. Another factor that may influence the sensitivity to EGFR inhibitors is the level of expression of the target itself, and also the presence of alterations in downstream signalling independent of receptor activation. For example, both the HCC1937 [19] and MDA-MB-468 cells [39] are PTEN null, resulting in increased propagation of the PI3-kinase pathway. She *et al*. have previously shown that by inhibiting the PI3-kinase pathway with LY294002 they can sensitize cells to Iressa [30], and we also found that by suppressing the expression of YB-1, which is downstream of phospho-Akt [10], using siRNA in the HCC1937 cells we were able to increase the effect of Iressa. Why YB-1 sensitizes BLBC cells to Iressa is an interesting question. YB-1 has been shown to regulate the *MDR1 *gene [40,41], and thus the P-glycoprotein pump, a member of the ABC family of transporters. This pump is involved in the efflux of many drugs, and has been associated with resistance to many chemotherapeutic agents [42]. We recently performed a ChIP on chip analysis of YB-1 target genes in SUM149 cells, and identified ~15 ABC transporter family members that were putatively bound by YB-1, including ABCG2, ABCA5 and ABCC3. Studies carried out by Özvegy-Laczka *et al*. showed that multidrug transporters such as ABCG2 may be involved in the resistance to tyrosine kinase inhibitors such as Iressa by modulating the uptake and extrusion of these drugs to and from cells [43]. In fact, they specifically show that ABCG2, but not mutant ABCG2, protects the lung cancer cell line A431 from Iressa-induced growth inhibition [44]. A more recent study [45] also confirms these findings with the demonstration of decreased intracellular accumulation of low concentrations of Iressa (0.1 μM to 1 μM) and higher efflux with 1 μM Iressa. Although further work is required to ascertain the mechanism involved, the suppression of YB-1 expression could indirectly increase the levels of these inhibitors in the cells, allowing them to bind to their target and reduce cell growth.

Not withstanding that SUM149 cells are sensitive to Iressa, suggesting that some BLBCs may be also, we recognize that acquired resistance to inhibitors such as Iressa is a common problem. There are many studies that implicate the overactivation of alternative signalling pathways, such as the insulin-like growth factor 1 pathway [46] and MET receptor amplification, leading to the activation of ERBB3–Akt pathway [46]. Alternatively, downstream pathways can become constitutively activated, an example being KRAS, which has been reported in lung and colon cancers [47-50]. Given this problem of acquired resistance, and the fact that many BLBC cases will not be sensitive, using Iressa in combination with an inhibitor for a downstream component may provide more long-term benefits.

Although we have established an association between YB-1 and EGFR in BLBC, it is likely that this transcription factor regulates the expression of other proteins linked to BLBC. For example, YB-1 regulates proliferating cell nuclear antigen (PCNA) and topoisomerase IIα [51], both of which are expressed in BLBC [52]. In colorectal carcinomas, YB-1 and topoisomerase IIα are co-ordinately expressed [53]. Likewise, similar expression patterns are reported in lung cancer [54] and synovial sarcomas [55]. More direct evidence for this association is supported by Shibao *et al*. who reported that knocking down YB-1 with antisense attenuates topoisomerase IIα reporter activity [53]. These and other YB-1 target genes are yet to be confirmed in BLBC. If *PCNA *and topoisomerase IIα are YB-1-responsive genes in BLBC, it would explain why the expression of this transcription factor is clearly associated with poor survival, based on work previously done by us [4] and others [56]. There are currently no commercially available inhibitors to YB-1. However, as YB-1 transactivates many growth-promoting genes, and we have shown that it can increase sensitivity to approved agents in BLBC, the question of whether it would also be a potent therapeutic target for this aggressive type of breast cancer is being actively pursued in our laboratory.

## Conclusion

We conclude from our data that YB-1 has a role in *EGFR *gene expression in BLBC. Furthermore, we demonstrate that tumour cell growth can be attenuated by blocking *EGFR*, alone or in combination with YB-1 inhibition, providing new possibilities for the treatment of this highly aggressive disease.

## Abbreviations

BAC = bacterial artificial chromosome; BLBC = basal-like breast cancer; CGH = comparative genomic hybridisation; ChIP = chromatin immunoprecipitation; ck5/6 = cytokeratin 5/6; DMSO = dimethyl sulphoxide; EGFR = epidermal growth factor receptor; EMSA = electrophoretic mobility shift assay; ER = estrogen receptor; HER2 = human epidermal growth factor receptor 2; IHC = immunohistochemistry; PR = progesterone receptor; SMRT = submegabase resolution tiling; TMA = tumour tissue microarray; YB-1 = Y-box binding factor-1; YRE = YB-1 responsive element.

## Competing interests

The authors declare that they have no competing interests.

## Authors' contributions

ALS carried out the luciferase assays, EMSA, phospho-YB-1 ChIP, growth assays and soft agar and was involved in drafting the manuscript. GH carried out the TMA, AA carried out the western blots on HCC1937 cells, HJ carried out the ChIP, KH was involved in acquisition of data for the growth assays, EP carried out the western blot characterising the SUM149 and HCC1937 cells, AS, TPHB and WL performed the array CGH, TON was involved in the TMA, UK and PRM made the phospho-YB-1 antibody, SA provided the primary BLBC tissue and SED conceived the studies and was involved in drafting the manuscript.

## Supplementary Material

Additional file 1 A table showing PCR primers for 28 exons of *EGFR*. Forward primer sequences were prefixed with a 21M13 sequencing tag, TGTAAAACGACGGCCAGT and reverse primer sequences were prefixed with an M13R sequencing tag, CAGGAAACAGCTATGAC. The primers (21M13 and M13R) were then used in the corresponding sequencing reaction. Click here for file

Additional file 2 A table showing sequence analysis of *EGFR *from the SUM149 cells. Variants were identified in exons 1, 12, 13, 15, and 20. The variants in exons 12, 13, 15 and 20 relate to SNPs that have been previously reported for EGFR. Click here for file
