# Supplementary material for: Epidermal growth factor receptor (EGFR) is transcriptionally induced by the Y-box binding protein-1 (YB-1) and can be inhibited with Iressa in basal-like breast cancer, providing a potential target for therapy
Source: Breast Cancer Res. 2007 Sep 17;9(5):R61. doi: 10.1186/bcr1767 (PMC2242657; doi:10.1186/bcr1767)
Supplement: Additional file 1 — A table showing PCR primers for 28 exons of EGFR. Forward primer sequences were prefixed with a 21M13 sequencing tag, TGTAAAACGACGGCCAGT and reverse primer sequences were prefixed with an M13R sequencing tag, CAGGAAACAGCTATGAC. The primers (21M13 and M13R) were then used in the corresponding sequencing reaction. [file bcr1767-S1.pdf]

Table 1 supplemental. **Sequencing primers**

Exon	Annealing Temperature, °C (T_{ann})	Forward Primer Sequence	Reverse Primer Sequence	Product Length (with seq tags)
1	60	tcgcattctcctcctcctct	cgcagctgatctcaaggaa	622
2	57	tggaccttgagggattgttt	ccaggcctttctccacttag	329
3	59	tcgtgtgcattagggttcaa	ttctccgaggtggaattgag	428
4	59	tgcatccttcatgggaattt	cccagtgctgtagagctgtc	300
5	60	agccagccaaacaatcagag	aactgcatgcggtgagattt	412
6	60	catgaaaaagtctgcaagtgct	aagtcttctgtcctggtgtgg	322
7	60	gctttctgacgggagtcaac	aggagacagagcgggacaag	334
8	58	ccatcacccctcaagaggac	gaggaagatgtgttcctttgg	333
9	55	gcctgtggatccctagctatt	ctgaaacaaacaacagggtga	331
10	55	gtcacaggttcagttgcttgt	gggaacaggaaatatgtcgaa	285
11	58	ccctgagagtctagagtaatgtctcat	tctcctgttaagcctaatttcca	280
12	56	tcaatcaaaggtggtctgga	aaatgggaatagcccttcaa	414
13	61	caaggtcatggagcacagg	aacaacaacctggagcctta	333
14	56	ggtgatttgtgttcctgcaa	tcatcactgttcggcttctg	262
15	60	atcatttggctttccccact	acaaacctcggcaatttgtt	407
16	56	caacatccagacacatagtgatttt	gtcagaaatgcaggaaagca	300
17	60	gccaaggccatggaatct	aactgctaatggcccgttct	322
18	60	gtgtcctggcacccaagc	ccccaccagaccatgaga	340
19	60	cagcatgtggcaccatctc	cagagcagctgccagacat	273
20	60	cattcatgcgtcttcacctg	catatccccatggcaaactc	412
21	60	agccataagtcctcgacgtg	acccagaatgtctggagagc	372
22	56	agactgaaatcccctgttgc	tcagtacaatagatagacagcaa tga	435
23	60	gaagcaaattgcccaagact	atttctccagggatgcaaag	413
24	58	gcaatgccatctttatcatttc	gctggcatgtgacagaacac	281
25	59	agacccctgctcctatagcc	ccatgtgagtttcactagatggtt	379
26	57	cctgcattcaggaaaagtgg	ggaaaaacccacacaggaag	284
27	56	caaggagatctcgggtga	gagatgctggagggagcac	329
28	62	atcctgcatgggatggtg	gtggcttggtcctgggtat	517
