# Supplementary material for: Epidermal growth factor receptor (EGFR) is transcriptionally induced by the Y-box binding protein-1 (YB-1) and can be inhibited with Iressa in basal-like breast cancer, providing a potential target for therapy
Source: Breast Cancer Res. 2007 Sep 17;9(5):R61. doi: 10.1186/bcr1767 (PMC2242657; doi:10.1186/bcr1767)
Supplement: Additional file 2 — A table showing sequence analysis of EGFR from the SUM149 cells. Variants were identified in exons 1, 12, 13, 15, and 20. The variants in exons 12, 13, 15 and 20 relate to SNPs that have been previously reported for EGFR. [file bcr1767-S2.pdf]

Table 2 Supplemental

EGFR Variants Detected in SUM149 Cell Line

Exon #	Variants			Interpretation
	bp # within exon	bp # within transcript	Amino Acid #	
1	A56CC	A56CC	Not translated	SNP: rs712830
2	No variants			
3	No variants			
4	No variants			
5	No variants			
6	No variants			
7	No variants			
8	No variants			
9	No variants			
10	No variants			
11	No variants			
12	A19AG	A1563AG	A439A	SNP: rs17290005
13	G64AG	G1808AG	R521K	SNP: rs11543848
14	No variants			
15	G66AG	G2034AG	P596P	SNP: rs17290162
16	No variants			
17	No variants			
18	No variants			
19	No variants			
20	G78AG	G2607AG	Q787Q	SNP: rs17337198
21	No variants			
22	No variants			
23	No variants			
24	No variants			
25	No variants			
26	No variants			
27	No variants			
28	No variants			

These variants can be viewed using Ensembl:
http://www.ensembl.org/Homo_sapiens/transview?db=core;transcript=ENST00000275493
